# Training socially accountable clinician-citizens: integrating clinical public health education in a medical school curriculum

**DOI:** 10.1080/10872981.2025.2469972

**Published:** 2025-02-26

**Authors:** Kofi Essel, Hana Akselrod, Sonal Batra, Candice Dawes, Zareen Zaidi, Lawrence Deyton

**Affiliations:** a Department of Pediatrics, The George Washington University School of Medicine and Health Sciences, Washington, DC, USA; b Elevance Health, Indianapolis, IN, USA; c Department of Medicine, Division of Infectious Diseases, The George Washington University School of Medicine and Health Sciences, Washington, DC, USA; d Department of Emergency Medicine, The George Washington University School of Medicine and Health Sciences, Washington, DC, USA; e Department of Medicine, Division of General Internal Medicine, The George Washington University School of Medicine and Health Sciences, Washington, DC, USA; f Department of Medicine, The George Washington University School of Medicine and Health Sciences, Washington, DC, USA

**Keywords:** Clinical public health education, population health initiatives, community health engagement, social accountability in medical education, curriculum innovation in medical training

## Abstract

By adopting a holistic perspective that looks ‘upstream’ at the underlying determinants of health, physicians can develop more effective strategies for promoting wellness and reducing health inequities in an increasingly diverse and complex society. Public health focuses on disease prevention and promotion of health through organized efforts by individuals and society. Population health focuses on the health outcomes of a group of individuals. We designed the Clinical Public Health curriculum, a pedagogical framework designed at the George Washington University School of Medicine & Health Sciences that breaks down traditional silos between didactic public and population health teaching, patient care and community engagement for medical students. It aims to train socially accountable clinician-citizens through an integrated, longitudinal curriculum across the four years of medical school. In this article we describe one aspect of the curriculum – four self-contained ‘summits’ – which can be used as a template for others seeking to develop a curriculum focusing on social accountability and engagement with community and governmental partners. During these multi-day applied educational experiences, medical students engage with key stakeholders, community members, community-based organizations, and state and national agencies to develop innovative approaches to engage in advocacy and population health. Enhanced medical school curricula focusing on the development of socially accountable clinician-citizens is an urgent need to develop more meaningful clinical-community interventions, support professional development, put context on the impact of health-related social needs on patients and families, and transform healthcare delivery and policy through greater community connection and advocacy.

## Introduction

To practice medicine effectively in an increasingly diverse society and to prepare for roles physicians must take on to address ‘upstream’ factors that affect health inequities, medical students must be trained to understand social determinants of health and broaden their scope of practice to include addressing health promotion through advocacy and community engagement [1]. In 2000, the Association of American Medical Colleges and the Centers for Disease Control and Prevention partnered to award Regional Medicine-Public Health Educational Centers across the country to promote population health curricula in U.S. medical schools [2]. While it may appear that many medical schools have responded to this call [3] most provide population and public health exposure as isolated, short-duration courses of limited focus, with few providing options for longer immersion programs [4]. This fragmentation in public health education as well documented with other social sciences may attenuate the value of the topic in medical education, othering population health as a specialty rather than an integrated and core principle of meaningful healthcare [5]. Thereby decreasing the confidence, knowledge, and skills of future providers. Although not a regional award recipient, we share our experience integrating public and population health, health policy and community engagement into the curriculum since 2014 at the George Washington University School of Medicine & Health Sciences (GW) through our novel Clinical Public Health (CPH) curriculum. CPH integrates population health, public health, health policy, and community engagement in the MD program. Its goal is training medical students to be ‘clinician-citizens,’ a model which charges medical professionals to improve systems of care and population health through advocacy and community participation [6]. In addition to required coursework at GW, ‘Summits’ are the hallmark of the CPH curriculum [7]. These ‘Summits’ are composed of applied educational experiences using experiential learning frameworks that comprise a series of unique multi-day real-world collaborative field projects that are integrated into the GW clinician education requirements building on clinical and public health curricula throughout the four years of medical school [8]. GW is one of the few US schools of medicine to integrate comprehensive public and population health throughout the preclinical and clinical curriculum for all students.

GW is located in the heart of Washington D.C. in proximity to a number of state/federal agencies, public health and policy resources. Through four uniquely designed summits in the MD program, students build foundational knowledge and skill-building in public health and population health, public health research techniques, critical and creative thinking, teamwork and leadership skills, community engagement, and translation of knowledge into action (Table 1). In this article we illustrate how the curriculum, summits, and community partnerships have shaped CPH education at our institution. The CPH curriculum and our ongoing community partnerships contribute to preparing our students to meet the challenges of a rapidly changing healthcare landscape and to play a leading role in addressing the complex factors that drive health inequities. Key features of this curriculum can be adapted by other medical schools to develop an integrated approach to public health in medical education, shaping the next generation of physicians to be equipped to improve both individual patient outcomes and population health on a broader scale.

**Table 1.** Description and examples of clinical public health summit student projects.

Summit Topic & Objectives	Assignment	Example Project	Selected Student Feedback
Summit 1: How Clinicians Can Help End the HIV Epidemic **Objectives**: Discuss the socio-cultural context in which HIV/AIDS occurs, the challenges of Ending the HIV Epidemic in the US, and the role that clinicians can play in eradicating it. Identify opportunities for HIV prevention and early intervention to promote the health of individuals and diverse populations. Compare and contrast the state and local epidemiology and health systems for the HIV/AIDS epidemic among the identified jurisdictions. Identify current policies and programs supporting population-level health outcomes for the identified HIV/AIDS domains and jurisdictions. Describe factors that contribute toward working effectively on teams with respect and collaboration in decision-making processes. Define learning objectives to guide research efforts, stakeholder input, and field visits for action plan development. Practice designing and presenting population-based interventions for HIV/AIDS that are evidence-based, culturally competent, and effectively utilize system and community resources and capacities for the jurisdiction.	Teams of 20–25 students develop and present a jurisdiction-specific action plan based on the National HIV/AIDS Strategy – Ending the HIV Epidemic – structured along the following domains: Diagnose all people with HIV as early as possible. Treat people with HIV rapidly and effectively to reach sustained viral suppression. Prevent new HIV transmissions by using proven interventions. Respond quickly to potential HIV outbreaks by providing prevention and treatment services. Each student team develops a written proposal to end the HIV epidemic for their assigned jurisdiction. The proposal represents a cohesive synthesis of the above four key strategies, reflects foundational knowledge of the medical science of HIV/AIDS, and builds upon known jurisdiction-specific responses to end the epidemic. Students are advised to make recommendations specific to the hardest-hit communities, and take into account how proposals fit into other public health efforts and address barriers to care and disparities in health outcomes. Each team crafts a written summary and presents it for a panel of experts who provide critiques. Evaluation: Students are evaluated on their final written proposals and verbal presentations as a group by key stakeholders and faculty. Evaluation rubric assesses for comprehensiveness, effectiveness, innovation, cohesiveness, and delivery of content.	A State Action Plan for Iowa proposing the expansion of confidential and accessible services for HIV diagnosis, treatment, prevention, and response by offering mail-based and mobile app-based programs for prevention and treatment of HIV, training community providers in counseling and harm reduction, and building a coalition of locally influential communicators on HIV.	**2023 HIV Summit Quantitative Feedback**: Evaluations were collected for the first semester of CPH curriculum, for which the HIV Summit serves as capstone. 91% (105/115) Medical students agreed or strongly agreed that they achieved the Learning Objectives for the CPH curriculum and HIV Summit. 58% (67/115) Medical students agreed there was sufficient integration between CPH and the basic science curriculum overall. Multiple student comments singled out the HIV Summit as a positive example of content integration. **Exemplary Medical Student Quotes**: *‘The HIV summit went beyond the scope of the classroom towards how things work in the real world. I have a better appreciation for the people tackling these issues and the constraints they have.’* *‘The HIV Summit was an incredible experience that allowed me to apply the knowledge I learned in the classroom to real-world settings. It was very meaningful to work as a team with a stakeholder to create a proposal to help increase HIV diagnoses and treatments in the city where I went to college.’*
Summit 2: How Clinicians Can Help Eliminate Childhood Asthma in Washington DC **Objectives**: Recognize the major determinants of and contributing factors for asthma. Discuss the challenges and opportunities for preventing and eliminating asthma in Washington, D.C. Describe major determinants of and contributing factors for asthma. Explain local pediatric asthma epidemiology and its connection to health care disparities. Recognize the challenges and opportunities for preventing and eliminating asthma in Washington, D.C. Understand family stressors that can be addressed to improve quality of asthma care. Identify current Washington, D.C. programming designed to address asthma prevalence, attacks, and/or treatment. Practice designing population-based interventions for childhood asthma in Washington DC that are evidence-based, culturally competent, and effectively utilize system and community resources and capacities. Develop an innovative idea to combat a specific aspect of childhood asthma in Washington, D.C.	Teams of 4–5 students develop clinical public health ideas using advocacy and/or innovation to decrease the burden of childhood asthma in Washington, DC. Teams choose one of the following deliverables: Craft a 1,200-word opinion piece (‘Op-Ed’) proposing a way to improve care for children with asthma living in Southeast Washington, DC that focuses on a defined sector (housing, school, after-school, healthcare). The top 3 are published by the DC Chapter of the American Academy of Pediatrics. Create a pitch for an innovative product/tool/application that will improve care for children with asthma living in Washington, DC. Submissions are submitted to a university-wide venture competition with cash prizes and mentorship from entrepreneurial experts. Evaluation: Students are evaluated on their final opinion piece or innovative proposal as a group by key stakeholders and faculty. Evaluation rubric assesses for comprehensiveness, effectiveness, innovation, cohesiveness, and delivery of content.	An Op-Ed entitled, ‘A Breath of Fresh Air: Utilizing Community Health Advocates (CHA) and Student Educators to Combat the Asthma Epidemic in Southeast Washington, DC Schools’ promotes an evidenced-based program with a two-pronged approach mobilizing CHAs for education promotion community-wide for children with asthma. https://www.aapdc.org/asthma/ ‘Asthguard’ innovation seeks to identify asthma allergens in the home of children with asthma.	2023 Asthma Summit Quantitative Feedback: Evaluations are collected for the course that the Asthma Summit falls under. Only qualitative feedback is collected on the Asthma’s Summits contribution to student learning. **Exemplary Medical Student Quotes**: ‘*It encouraged us to think critically about a real-world problem affecting our surrounding environments. It helps students connect with some of the issues their community faces and encourages us to become involved in helping community members. The Asthma Summit allowed us to collaborate with other students and to use our creativity and research skills to create a project to help reduce asthma in the D.C. area. This was a great opportunity to learn how to collaborate with our peers.’* *‘Physicians may need to reach out to the public in the future with health information that can help people, and I think that this exercise allowed students to build a unique skill set to communicate effectively with people in a macro scale.’* *‘By placing us in a position to examine a complex medical issue, exacerbated by healthcare disparities and [Social Determinants of Health (SDOH)], we could utilize the tools we had to try and create innovate solutions to the problems our patients face, as we should be expected to as practicing physicians.’*
Summit 3: How Physicians Can Engage Obesity with Tools of Health Equity & Empathy in Washington, DC **Objectives**: Discuss the clinical public health challenges of addressing, preventing, and reducing obesity rates in Washington, DC Describe major determinants and contributing factors of obesity through a health equity lens in Washington, DC Describe the role weight bias and obesity stigma play in the care and management of individuals and populations with obesity Apply research and field work to proposal development for addressing obesity at the individual, community, and policy levels Demonstrate effective communication in advocating for recommendations to organization, state agency, and public health leaders on effectively addressing obesity in Washington, DC	Teams of 30 students develop proposals using the health equity of obesity framework* to address real-world problems faced by community organizations and state agencies. This framework includes: Increasing healthy options (i.e., grocery stores, food hubs). Reducing deterrents to healthy behaviors (i.e., predatory marketing practices, safety concerns). Improving social and economic resources (i.e., federal programs, nutrition security). Building community capacity (i.e., community education, community collaboratives). Course directors meet with organizations and agencies prior to the summit to select topics. Students are advised to ensure their proposals directly address organization/agency requests, centralize health equity, incorporate the lived experiences of key stakeholders they interview and avoid weight stigma. Each team crafts a written summary and presents it for a panel of experts and representatives from partner organizations, who provide critiques. Final written proposals are forwarded to partner organizations and agencies. Evaluation: Students are evaluated on their final written proposals and verbal presentations as a group by key stakeholders and faculty. Evaluation rubric assesses for comprehensiveness, effectiveness, innovation, cohesiveness, and delivery of content.	In response to a request by the District of Columbia’s Special Supplemental Program for Women, Infants, and Children(WIC), students developed a policy, community, and clinical based intervention to support the uptake of WIC in children above the age of 1 year old to counteract the precipitous decline in participation.	**2023 Obesity Summit Quantitative Feedback**: 86%(114/132) Medical students agreed they achieved the Learning Objectives. 80%(106/132) Medical students agreed the obesity summit allowed them to place what they learned about basic sciences into a public/population health context. ***Exemplary Medical Student Quote***: *‘The activities at the Obesity Summit greatly contributed to my professional development. By actively participating in these* *activities, I acquired valuable knowledge, insights, and skills pertaining to the field of obesity. The presentations and* *workshops offered a platform to stay updated on the latest research, evidence-based practices, and innovative approaches in managing obesity. Engaging with experts and fellow professionals broadened my network, leading to valuable connections and potential collaborations. Moreover, engaging in discussions and sharing experiences with peers enhanced my critical thinking and problem-solving abilities.’*
Summit 4: Population Health Summit – Your Residency Choice and Your Career **Objectives** Identify population-based health problems relevant to a specific specialty using epidemiologic principles, health systems analysis, and evidence-based methods to interpret information about health needs. Formulate and propose tangible ways in which population health can be improved through actions by physicians and partners by integrating biomedical, clinical, epidemiological, and social-behavioral sciences. Work collaboratively as a member of a team and apply self-directed learning strategies. Demonstrate an expanded scope of practice for 21st century health systems, integrating roles as an excellent clinician, public health and community health leader, and physician citizen.	Working individually or as small teams, students select and research a clinical public health-related topic in their chosen medical specialty and develop an ‘Action Plan’ to address, improve, remediate, or advance their chosen topic. Students select a topic at the end of third year and complete this project over the course of their final year of medical school, developing a written Action Plan using a provided template. Students orally present their projects to third year medical students and chairs and leaders in their chosen specialty fields to gain feedback and stimulate ideas for the next group of students. Evaluation: Students are provided feedback on their project presentation by faculty members in their planned medical specialty. Evaluation asseses for clarity, relevance, stakeholder engagement, and adherence to proposal template.	A team of students entering Anesthesia and Internal Medicine conducted a systematic review of the literature on safe opioid disposal methods. They interviewed institutional stakeholders (hospital Chief Quality Officer, hospital Director of Pharmacy, a general surgeon, a pain management physician, and several patients from a chronic pain clinic). They interviewed community stakeholders (2 community pharmacists and the Medical Director of a local community health center). Using this, they developed a proposed hospital protocol for safe disposal of opioids. They submitted finding as an abstract to a national conference and presented as a poster at the institutional research symposium.	**2023 Population Health Summit Quantitative Feedback**: 56% (55/98) of Medical students rated the summit as ‘Good’ or ‘Very Good’ on a 5-pt Likert scale. **Exemplary Medical Student Quotes**: *‘I enjoyed the faculty panel and hearing about how people have incorporated public health efforts into their careers. I also found the student panel really helpful in terms of hearing about people’s experiences with their* *projects, their advice and tips. The flexibility and independence was nice for our brainstorming/workgroups.’* *‘I really liked the brainstorming group with the surgical attendings. They provided a lot of great feedback and were* *truly collaborative in helping us flesh out our ideas.’*

*Obesity Equity Framework designed by Dr. Shiriki Kumanyika from the University of Pennsylvania School of Medicine [9].

## Description of summits

The topics of the four summits (Table 1) were chosen with input from community stakeholders and the educational and experiential objectives were designed by expert faculty based on their local practice experience and analysis of public health data for the region. Each summit is coordinated by a clinical public health trained physician faculty member with topic expertise, in addition to having oversight from the Associate Dean of Clinical Public Health. This course is part of the mandatory curriculum for the entire class of approximately 180 students throughout their 4 years of medical school and successful completion is required for graduation. The first two summits occur in Medical School Year 1 (MS1) the third summit takes place between Medical School Year 2 (MS2) and Medical School Year 3 (MS3) and the fourth summit is a longitudinal year-long project culminating in spring of Medical School Year 4 (MS4). The policy, academic, and community based organizations and stakeholder volunteers are selected through physician and faculty leaders and partners. Allowing for a diverse regional experience for medical student learners. In addition, throughout the curriculum students are able to work alongside other public health professional students, patient advocates, and key stakeholders ensuring a more robust interdisciplinary learning opportunity. Lastly, medical students are evaluated using a series of quantitative and qualitative surveys, written reports, oral presentations, and professionalism. Medical students are required to pass this course to matriculate in their medical education, and these data are also used to iterate the design of the curricula each year to better meet the learners needs.

## Summit one: how clinicians can help end the HIV epidemic - prescriptions for state and city action plans

The HIV Summit takes place over the course of 3 days at the end of MS1 fall semester. It is organized in partnership with the National Alliance of State and Territorial AIDS Directors (NASTAD) and features guest speakers including senior national leaders on HIV/AIDS policy, state HIV/AIDS directors, local leaders in HIV care and research, and patient advocates representing communities most affected by HIV (Table 2). It is constructed in the model of a three-day scientific, multidisciplinary conference, structured around key tenets of federal strategy including the ‘Ending the HIV Epidemic’ initiative and the National HIV/AIDS Strategy. The HIV Summit also serves as the capstone project for the first semester of the CPH curriculum.

**Table 2.** Stakeholders and partners.

Stakeholders and partners participating in the GW-NASTAD Clinical Public Health Summit on HIV (2014–2021)		
Category	Selected Past Participants	
Community	Prevention Access Campaign	
	Capital Trans Pride	
	Casa Ruby	
	Community Education Group	
	National Alliance of State & Territorial AIDS Directors	
	Community Advisory Board for the DC Center for AIDS Research	
Health Care	GW Medical Faculty Associates	
	GW Clinical Trials Unit	
	DC Veterans Administration Medical Center	
	Whitman Walker Health	
	Family and Medical Counseling Services	
	Unity Health Care	
	Providence Hospital	
Academic	DC Center for AIDS Research	
	GW School of Medicine and Health Sciences	
	GW Milken Institute School of Public Health	
	Georgetown University	
Government	Congressional HIV/AIDS Caucus, Department of Health and Human Services – Office of the Assistant Secretary for Health, and HIV/AIDS Directors from multiple states/cities	
	US White House Office of National AIDS Policy	
	US National Institutes of Health, National Institute of Allergy and Infectious Diseases	
	US Health Resources and Services Administration, HIV/AIDS Bureau	
	US President’s Emergency Plan for AIDS Relief	
	DC Department of Health HIV/AIDS, Hepatitis, STD and TB Administration (DC is one of approx., 20 states that have participated over the years)	
**Stakeholders and partners participating in the GW Clinical Public Health Summit on Asthma (2018–2021)**		
**Category**		**Selected Past Participants**
**Community/Non-Profit Organizations**		American Academy of Pediatrics (DC Chapter)
		Asthma Home Visiting Program
		Children’s Law Center
		Yachad
		AmeriHealth Caritas District of Columbia
		Children’s School Services
**Health Care**		Child Health Advocacy Institute at Children’s National Hospital
		IMPACT-DC
**Academic**		Children’s National Hospital
		GW School of Medicine and Health Sciences
		GW Milken Institute School of Public Health
		GW New Venture Competition
**Stakeholders and partners participating in the GW Clinical Public Health Summit on Obesity (2014–2022)**		
**Category**		**Selected Past Participants**
**Local/Regional Community Organizations/Non-Profits**		American Heart Association (AHA) (Greater Washington Region)
		Arcadia Center for Sustainable Food and Agriculture
		Capital Area Food Bank (CAFB)
		DC Action for Children
		DC Central Kitchen
		DC Hunger Solutions
		Food & Friends
		FRESHFARM
		Martha’s Table (MT)
		Spaces in Action
		Summit Health Institute for Research & Education (SHIRE)
		The Green Scheme
		Town Hall Education Arts Recreation Campus (THEARC)
		Ward 7 Health Alliance Network
		Washington Nationals Youth Baseball Academy
		Young Men’s Christian Association (YMCA) of Metropolitan Washington
**National Non-Profits Organizations**		American Heart Association (AHA) (National)
		American Planning Association
		Build our Kids’ Success (BOKS)
		Center for Science in the Public Interest (CSPI)
		Common Threads
		Culinary Medicine Specialist Board
		Food Corps
		Food Research & Action Center (FRAC)
		Giant Grocery Store
		KABOOM!
		Mom’s Meals
		National League of Cities
		Obesity Action Coalition (OAC)
		Parks Rx America
		Partnership for A Healthier America (PHA)
		Playworks
		Rudd Center for Food Policy and Obesity
		Step Afrika!
		The Pew Charitable Trusts
		The Trust for Public Land
		Women and girls advancing nutrition dietetics and agriculture (WANDA)
Private Organizations		Giant Foods
		Lebanese Taverna
		Sodexo US
Health Care		AmeriHealth Caritas
		Burke Pediatrics, LLC
		Children’s National Hospital- Improving Diet, Energy and Activity for Life (IDEAL) Clinic
		Children’s National Hospital-Child Health Advocacy Institute (CHAI)
		DC Health Matters
		Fairfax County Health Department
		Georgetown University
		GW Medical Faculty Associates
		Holy Cross Health Network
		Mary’s Center
		Medstar Washington Hospital Center
Academic		CentroNía
		DC Bilingual Public Charter School
		GW Food Pantry-‘The Store’
		GW Milken Institute School of Public Health
		GW Office of Innovation & Entrepreneurship
		GW School of Medicine and Health Sciences
Government		Association of State and Territorial Health Officials (ASTHO)
		DC Department of Corrections
		DC Department of Health (DC Health)
		DC Food Policy Council
		District of Columbia Public Schools (DCPS)
		Fairfax County Health Department
		Food & Drug Administration (FDA)
		Office of the State Superintendent of Education (OSSE)
		The National Institutes of Health National Heart, Lung, and Blood Institute (NHLBI)
		The Special Supplemental Nutrition Program for Women, Infants, and Children (WIC)

The participants listed below are representative of local, regional, and national stakeholders across approximately eight years for Summits 1–3 with some variation based on availability and focus

The HIV Summit challenges the students to apply their medical knowledge and demonstrate their skills as physician-advocates by working in groups to craft original proposals (Table 1) addressing the HIV epidemic at the level of their assigned jurisdiction (state or major city). On the last day, each proposal is presented by student group representatives and critiqued by a panel of experts including NASTAD leadership, senior public health faculty, and high-level officials from the U.S. Department of Health and Human Services.

## Summit two: how clinicians can help eliminate childhood asthma in Washington, DC

The second summit focuses on asthma and spans 3-days during MS1 (Table 1). This summit is a collaboration between Children’s National Hospital’s program Improving Pediatric Asthma Care in the District of Columbia and our medical school. The summit highlights local disparities related to asthma and how future clinicians can help eliminate childhood asthma in Washington, DC.

As part of the experience, students work in groups to develop proposals which focus on prevention strategies that address childhood asthma prevalence, exacerbations, and/or treatment.

## Summit three: how physicians can engage obesity with tools of health equity & empathy

The summit on obesity is the third in the series with intermittent events that span 3 months beginning in MS2 (Table 1). In the first phase, students learn about the clinical and population level dimensions of obesity through implicit weight bias assignments and expert and patient lectures. In phase two, local community-based organizations and state agencies propose real world challenges (Table 2) for students to solve working alongside the organizations’ leaders. Students engage with key stakeholders, community members, and local and national experts. The culminating experience requires students to present proposals to their peers, community organizations, and state agency representatives.

## Summit four: population health: how physicians can help improve population health in every specialty

The Population Health Summit spans 12 months, commencing in a 2-day kickoff experience at the end of the MS3 year, and culminating in a final presentation at the MS4 conclusion.

Unlike the established topics of the previous summits, in this summit students self-select a CPH-related topic that relates to their intended medical specialty and then develop and propose an action plan individually or in small groups utilizing best practices in public health to identify a clear problem, target population, relevant stakeholders, and evaluation metrics for their proposal (Table 1). There is an additional wellness and resilience component to this summit which is not discussed in detail in this article.

## Conclusion

Now more than ever, it is important to graduate ‘clinician-citizens’ who are prepared to deliver excellent patient care in 21^st^-century health systems and who can identify and address important health system-, community- and policy-level issues that impact the health of individuals and diverse populations. The cutting-edge nature of our summit curriculum has caught the attention of medical schools, physicians, and public health experts [10]. There has been growing interest in integrating public health into medical education [11–14]. However, students often report feeling disillusioned, disengaged, and disappointed with public health curricula [15,16]. Students overwhelmingly report positive experiences with the summits and indicate that this focus on Clinical Public Health was a major contributor to selecting the medical school. In addition, community organizations and state agencies express appreciation for the opportunity to train medical students, annually return to join our curriculum, and regularly use the novel thought leadership in organizational proposals, grants, and interventions. Factors which promote student engagement in our curriculum include (1) application of their clinical and public health learning to work on real-world community health problems, (2) formation of community partnerships, and (3) guidance by faculty from diverse backgrounds with public health experience who serve as citizen-clinician role models for our students. The process of including higher level Kirkpatrick program evaluation for CPH has been noted to be challenging [17]. At GW we are endeavoring to collect data related to graduating students’ expanded scope of practice, as part of their professional toolkit regardless of specialty choice. We also continue to work on improving the integration of public health with clinical experience in the curriculum. Although our experiences are unique due to proximity of the federal government and other agencies, they may readily be replicated at other institutions by identifying and listening to community stakeholders concerning areas of possible collaboration, identifying local physician champions and faculty members with unique expertise in particular clinical public health topics of local interest, engaging with state and local jurisdictional public health agencies, and intentionally identifying curricular time to integrate training into medical school curriculum. Longitudinal research examining the career trajectories and community engagement of graduates exposed to this innovative curriculum, compared to those from traditional medical education programs, would provide critical insights into the approach’s long-term effectiveness. Furthermore, investigating the curriculum’s adaptability across diverse institutional settings and evaluating the influence of faculty public health training on student performance could generate valuable evidence to advance medical education strategies. Limitations of our study include data collection at a single urban institution and access to faculty with public health backgrounds. As the program grows we plan to undertake higher-level program evaluation to address the impact of the program.

Lastly, we would like to point out that the term ‘clinical public health’ has been coined by our institution to reflect the integration of public and population health with clinical training. This has been in response to the critique of public and population health as being siloed and isolated from real life community problems. Since launching the CPH curriculum and Summits, we have continuously received enthusiastic positive feedback from students as well as stakeholder participants. These affirmative experiences reported by students and community organizations highlight the acceptability of these less fragmented and more integrated curricula for public health in medical education. Future clinicians will find themselves engaging in food/nutrition insecurity, housing insecurity, and patient transportation challenges inside the clinical encounter, but with this knowledge and skill building students may feel more equipped to take the opportunity to impact change outside of the four walls of clinical settings. Therefore, we would encourage other educators to explore clinical public health as a pedagogical approach to help develop clinician-citizens and to share their experiences with the medical education community, aiming to ultimately not just provide our students with better learning experiences but to also to prepare them for their future roles in society. Medical educators and institutions play a crucial role in transforming how future physicians perceive their responsibility and their ability to engage in healthcare. Clinical public health must be envisioned not as a specialty practice for a few physicians but as an core institutional necessity for each and every future physician.
